# Breast cancer phenotypes in carriers of pathogenic *POT1* variants

**DOI:** 10.1007/s10689-026-00589-6

**Published:** 2026-07-10

**Authors:** Tal Hadar, Aasem Abu Shtaya, Rinat Bernstein-Molho, Marina Eskin-Schwartz, Inbal Kedar, Tamar Peretz-Yablonsky, Shiri Shkedi-Rafid, Rinat Yerushalmi, Aviad Zick, Gili Reznick-Levi, Tanir Allweis, Yael Goldberg

**Affiliations:** 1 Department of Breast Surgery, Hadassah Medical Center, Jerusalem, Israel; 2 Faculty of Medicine, Hebrew University, Jerusalem, Israel; 3 Unit of Gastroenterology, Lady Davis Carmel Medical Center, Haifa, Israel; 4 Susanne Levy Gertner Oncogenetics Unit, The Danek Gertner Institute of Human Genetics, Sheba Medical Center, Ramat Gan, Israel; 5 Gray faculty of Medical and Health Sciences, Tel Aviv University, Tel Aviv, Israel; 6 Genetics Institute, Soroka Medical Center, Beer-Sheva, Israel; 7 Faculty of Health Sciences, Ben Gurion University, Beer-Sheva, Israel; 8 Recanati Genetics Institute, Rabin Medical Center, Petach Tikva, Israel; 9 Sharett Institute of Oncology, Hadassah Medical Center, Jerusalem, Israel; 10 Department of Genetics, Hadassah Medical Center, Jerusalem, Israel; 11 Davidoff Cancer Center, Rabin Medical Center, Petah Tikva, Israel; 12 Genetics Institute, Rambam Health Care Campus, Haifa, Israel

**Keywords:** POT1, Hereditary breast cancer, Germline pathogenic variants, Telomeres

## Abstract

Germline pathogenic variants (PVs) in *POT1*, one of the shelterin complex genes, correlate with tumor predisposition, primarily with melanoma, hematologic malignancies, sarcoma, papillary thyroid carcinoma and glioma. Breast cancer (BC) risk has not been shown to be elevated. We analyzed BC occurrence and features in a cohort of 29 female PV heterozygotes, of whom 13/29 (45%) were diagnosed with BC. Data regarding genetic, clinical, pathologic, treatment, and outcome characteristics were extracted. Patients in our cohort harbored three different POT1 PVs; The c.233T > C Ashkenazi founder PV occurred in 11/13 (84.6%). Median age at first BC diagnosis was 54 years (range 44–72); no patient was diagnosed before the age of 40. Pathological subtypes varied; invasive ductal carcinoma was the most common. All primary tumors were estrogen receptor positive; one was HER2-enriched; no triple-negative cancers were observed. Stage at diagnosis varied: 6 of 10 tumors with known staging were stage 0 or I, and one patient presented with metastatic disease. Treatment approaches were diverse as clinically appropriate. After a median follow-up of 110 months, three second BC events occurred, with no BC-related mortality. Personal and family history of other malignancies were frequent. This is the first dedicated report describing BC phenotypes in *POT1* PV heterozygotes. Our findings suggest that enhanced BC surveillance may be warranted in this population. Larger cohorts are needed to further characterize the clinicopathological features of BC in *POT1* carriers, to define lifetime BC risk and determine whether BC-specific screening recommendations should be established for this group.

## Background

Telomeres protect chromosomes ends, consist of repetitive nucleotide sequences, and shorten with each cell cycle. Dysfunctional telomeres may lead to inefficient DNA damage repair, genomic instability and tumorigenesis [1]. Telomeric DNA is bound by the shelterin protein complex, which is involved in maintenance of its structure and length regulation [2], and the Protection of Telomeres 1 *(POT1)* gene encodes one of the shelterin protein subunits [3–5].

Pathogenic variants (PVs) in the *POT1* gene can cause telomere elongation and are associated with diverse phenotypes; telomere elongation has been associated with *POT1* tumor predisposition syndrome (*POT1*-TPD) [3]. Constitutional *POT1-*related tumor types primarily include cutaneous and uveal melanoma, sarcoma, chronic lymphocytic leukemia (CLL) and other hematologic malignancies, glioma/meningioma, papillary thyroid carcinoma (PTC) [4, 6–13]. A recent study described 23 cancer patients with *POT1* germline PVs. Index cancers were diagnosed at a median age of 50 years, and the disease spectrum included hematological malignancies, papillary thyroid, colorectal and lung cancer, glioblastoma, and endocrine tumors [14].

Recently published UK practice guidelines estimated lifetime melanoma risk at 3–5% with a wide range of age at diagnosis. Angiosarcoma risk was approximately 2%, and CLL was 1–2%, with overall penetrance lower than in *TP53*–related Li-Fraumeni syndrome (LFS). Recommendations include a baseline full body skin examination at diagnosis, discussion of risk and symptom awareness, frequency of follow-up and routine cardiac MRI based on individual and family history. Evidence strength was considered moderate. The overall lifetime cancer risk for individuals with a PV in *POT1* is unclear. Consistent with this, the UK guideline panel judged current evidence insufficient to warrant breast-specific surveillance outside of research. Notably, the guidelines focused on the above-mentioned tumors and did not address follow-up for other malignancies that have been associated with *POT1* PV heterozygotes [15].

Hereditary Breast Cancer (BC) is mainly associated with germline PVs in high-penetrance DNA repair genes such as *BRCA1/2*, *PALB2*, *PTEN* and *TP53*, and PVs in moderate penetrance genes such as *ATM* and *CHEK2*, which carry a higher-than-average risk for BC. Currently, BC risk is not considered to be elevated in *POT1* PV heterozygotes nor is it considered part of the *POT1*-TPD. Several *POT1* polymorphisms have been linked to increased risk of BC, however, these associations was not statistically significant [15]. In a literature review on *POT1* PV carriers and cancer risk, Andreotti et al. did not mention any reports of elevated BC risk [16].

Abu Shtaya et al. recently reported on a cohort of *POT1* PV carriers, in which five of eight (63%) women harboring the *POT1* Jewish Ashkenazi founder PV c.233T > C developed invasive breast cancer (age 44–67 years) [17].

This study describes an unselected BC patient cohort with germline *POT1* PVs, focusing on genetic, clinicopathological, treatment and outcome characteristics.

## Patients and methods

This cohort includes 13 female *POT1* germline PV heterozygotes who were diagnosed with BC. A subgroup of the cohort has been described in the report from Abu Shtaya et al. [17]. Twelve patients underwent multi-gene panel testing, while one patient had cascade testing only. Demographic, genetic, clinical, pathological, treatment, and outcome data were extracted from electronic medical records. The study was conducted at Rabin Medical Center and Hadassah Medical Center and was approved by the institutions’ ethics committees (approvals RMC-no. 0847-22; HMC-no. 0339-20).

## Results

Thirteen women carrying a *POT1* PV were diagnosed with 14 primary BC events. Of these, 11 women (84.6%) carried the *POT1* c.233T > C variant; one carried the splice-site variant c.950–2 A > G, and one had a frameshift variant (c.1672dup). One patient was also heterozygous for the *MITF* c.1273G > A p.Glu425Lys PV (Table 1).

**Fig. 1:**
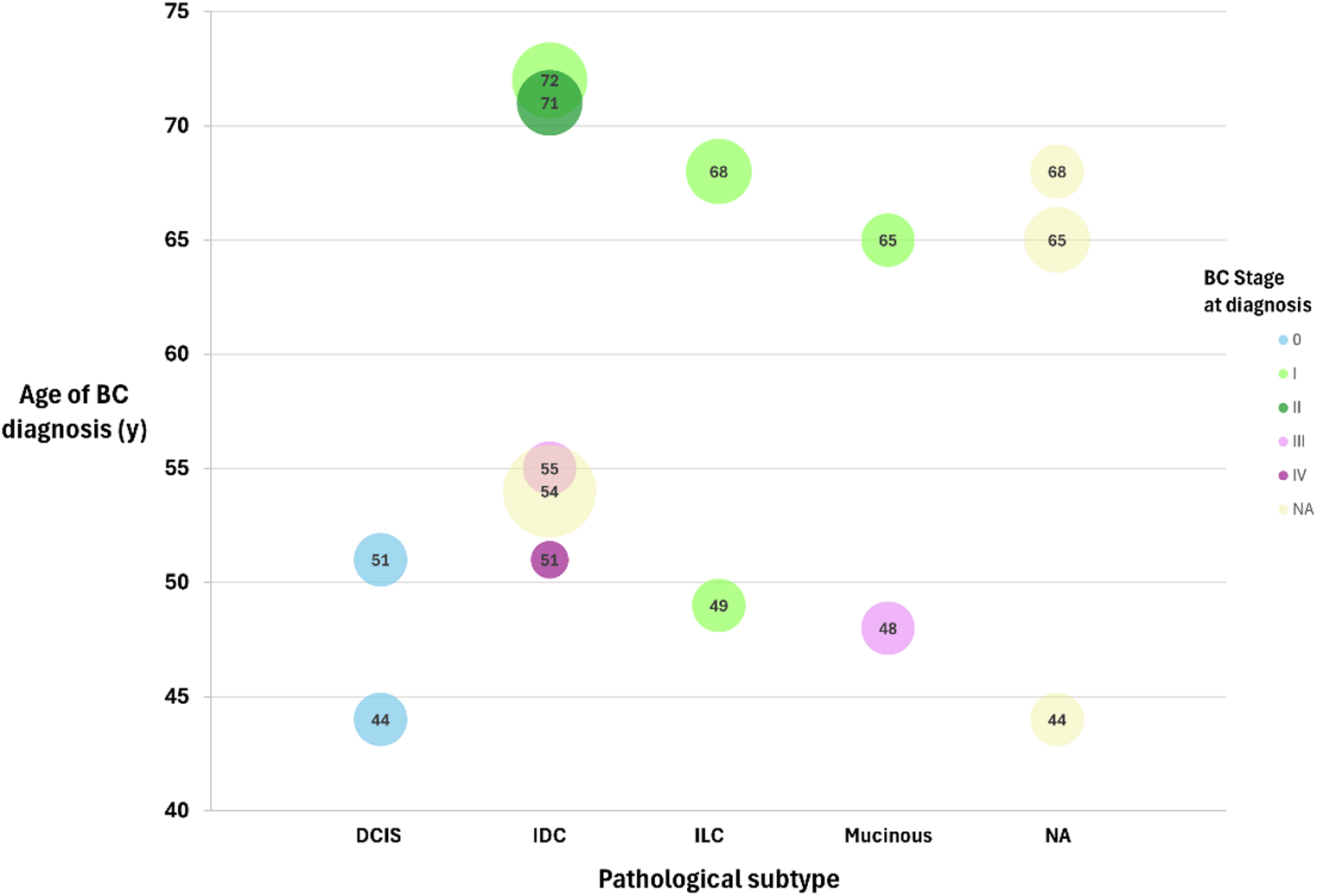
Breast cancer landscape in *POT1* PV heterozygotes. Bubble size according to number of additional cancers

Twelve of thirteen women had unilateral BC, and one had bilateral, non-synchronous BC. The median age at first breast cancer diagnosis was 54 years (range 44–72). None of the patients was diagnosed with BC before age 40. In four women, the first BC event (31%) occurred before age 50, in four between 50 and 59, and in five between ages 60 and 72 (Fig. 1).

Among the 10 patients with available pathological information for primary BC events, invasive ductal carcinoma (IDC) occurred in 5 cases, and invasive lobular carcinoma (ILC), mucinous carcinoma and pure ductal carcinoma in situ (DCIS) in two cases each. One patient with DCIS had a synchronous contralateral lumpectomy due to atypical ductal hyperplasia (patient 4). All tumors were estrogen receptor (ER) positive, one of these had low ER expression (10% of cells). One primary tumor was HER2-enriched. No cases of triple negative BC were observed in this case series. Stage at presentation varied: two patients were diagnosed with stage 0 (DCIS), four with stage I, one with stage IIA, two with stage IIIA (stages II and III with axillary lymph node involvement), and one patient had bone metastases at the time of diagnosis (stage IV) (Fig. 1). In six patients, the diagnostic modality was known. Of these, three presented with clinical symptoms, including a breast mass or nipple retraction.

Of the 8 patients with available surgical information, five underwent breast-conserving surgery and three had mastectomies. None had contralateral risk reducing mastectomy; one patient did not have surgery due to metastatic disease at presentation. In patients with invasive disease, 3 of 7 received chemotherapy. Adjuvant radiotherapy was administered following all breast-conserving procedures.

The median follow-up was 110 months (in 7 patients with available data). Three patients were diagnosed with a second BC event. One patient developed contralateral primary BC with different disease characteristics 12 years after the initial diagnosis (patient 8); In another patient, ipsilateral chest wall recurrence occurred 81 months post-surgery (patient 6). One patient had tumor recurrence to the skin and contralateral lymph nodes 6 years after initial diagnosis. The disease was ER negative, HER2-enriched. She was treated with pre-operative chemotherapy and HER2-targeted therapy and underwent bilateral mastectomy with a complete pathological response in the surgical specimen (patient 1). No breast-cancer-related deaths were observed.

Personal history of other tumors was frequent: 11/13 patients had at least one other malignancy, with a total of 20 non-breast tumors (average 1.5 additional tumors per patient, range 0–5). The most frequent tumors were melanoma (5 cases), followed by PTC (3 cases), sarcoma (2 cases, including one patient with two sarcoma events), and chronic lymphocytic leukemia (CLL, 2 cases). One patient was diagnosed with mucinous carcinoma of the ovary at age 30, 18 years prior to BC diagnosis. Melanoma was diagnosed in 5 cases; it occurred prior to BC in 3 of them, and in one case the tumors were synchronous. Non-*POT1* TPD syndrome cancer types in BC patients included hepatocellular carcinoma and bladder cancer, in one patient each.

In patients with available information, two (20%) reported a significant family history of BC: one had a mother, sister and maternal cousin with BC (patient 1), another patient reported two maternal aunts and a niece with BC (patient 8), all premenopausal at diagnosis. *POT1* status for these relatives was unknown. Melanoma was also reported in both families. In one family (patient 2), the patient’s maternal grandmother and aunt had ovarian and pancreatic cancer, respectively. Other cancer types documented in families of BC patients included non-small cell lung cancer, colorectal carcinoma and cholangiocarcinoma.

**Table 1:** Breast cancer characteristics and treatment in *POT1* PV heterozygotes

Patient #	PV	Stage	Pathology	ER / PR / HER2	Breast surgery	Chemo- therapy	Personal history of cancer (age, y)	Family history of BC	Family history of cancer	Recurr-ence	f/u (m)
1	c.233T > C	I	ILC	pos / pos / neg	BCS	No	melanoma (58), CLL (64), **BC (68)**	sister, mother, male cousin	Melanoma, gastric	DR	81
2	c.233T > C	NA	IDC	NA	mastectomy	No	PTC (40), melanoma (48), **BC (54)**, sarcoma (54,64), MDS (76)	none	Prostate, ovary, pancreas, lung	NA	155
3	c.233T > C	0	DCIS	pos / pos / NA	BCS	NA	melanoma (35), **BC (44)**	none	Melanoma, CRC	NA	NA
4	c.233T > C	0	DCIS	NA	BCS	NA	**BC (51)**, melanoma (51)	NA	NA	NA	NA
5	c.233T > C	I	ILC	pos / pos / neg	NA	NA	**BC (49)**, melanoma (60)	NA	Melanoma, CLL, CRC	NA	NA
6	c.233T > C	IIIa	mucinous	pos / pos / neg	mastectomy	Yes	mucinous ovarian carcinoma (30), **BC (48)**	none	CRC, gastric, lung	LR	198
7	c.233T > C	NA	NA	NA	NA	NA	**BC (65)**, HCC (70), bladder (70)	NA	NA	NA	NA
8	c.950–2 A > G	IIIa, I	IDC, mucinous	pos / pos / neg pos / neg / neg	BCS	Yes, No	**BC (55**,** 65)**	niece, maternal aunts	Melanoma, HL, cholangio-carcinoma	NA	236
9	c.233T > C	I	IDC	pos / pos / neg	BCS	No	sarcoma (62), **BC (72)**, GIST (73), solitary fibrous tumor	none	Sarcoma	NA	57
10	c.1672dup	II	IDC	pos / neg / NA	mastectomy	No	CLL (62), PTC (69), **BC (71)**	none	CLL, lung	NA	110
11	c.233T > C	IV	IDC	pos / neg / pos	none	Yes	**BC (51)**	none	Melanoma, leukemia, lymphoma	NA	6
12	c.233T > C	NA	NA	NA	NA	NA	PTC (31), **BC (44)**	none	Melanoma, sarcoma	NA	NA
13	c.233T > C	NA	NA	NA	NA	NA	**BC (68)**, MM (80)	none	Hystiocytoma	NA	NA

y−years, m−months, ILC−Invasive Lobular Carcinoma, IDC−Invasive Ductal Carcinoma, DCIS−Ductal Carcinoma In Situ, ER−Estrogen Receptor, PR−Progesterone Receptor, HER2−Human Epidermal growth factor Receptor 2, BCS−Breast Conserving Surgery, CLL−Chronic Lymphocytic Leukemia, PTC−Papillary Thyroid Carcinoma, MDS−Myelodysplastic Syndrome, HCC−Hepatocellular Carcinoma, GIST−Gastrointestinal Stromal Tumor, MM−Multiple Myeloma, CRC−Colorectal Carcinoma, HL−Hodkin’s Lymphoma, DR−distant recurrence, LR−local recurrence, NA−not available

## Discussion

*POT1*-TPD is associated with core malignancies such as melanoma, sarcoma, CLL, PTC and glioma.

A clear association between *POT1* PVs and BC has not yet been established. In our cohort of *POT1* heterozygotes, BC was observed in 13 of 29 (45%) women, a frequency comparable to that seen in other hereditary BC syndromes. To our knowledge, this is the first report to suggest a possible association between *POT1* PVs and BC, providing a detailed description of the clinical and pathological features in affected carriers.

The c.233T > C Ashkenazi founder allele predominated in the cohort, accounting for 11 of 13 cases. This missense variant has previously been reported in familial melanoma clusters [18]. This may suggest that the c.233T > C variant contributes disproportionately to BC risk in *POT1* carriers and warrants further investigation in larger cohorts. None of the patients were heterozygotes for other BC related PVs.

In our cohort, age at diagnosis of BC (median 54 years) was younger than reported for the general population (median 63 years) [18]. However, it was older than the median age of BC diagnosis among patients with PVs in the major BC susceptibility genes (including *BRCA1*, *BRCA2*, *CHEK2*, *PALB2* and *ATM*), reported to be 48 years [19], and notably older than that reported for *BRCA1* and *BRCA2* PV carriers (44 and 48 years respectively) [20]. Other BC characteristics in this *POT1* PV cohort appeared similar to sporadic BC with varied pathological features and stages at presentation. Three patients developed second BC events during follow-up, including one contralateral primary BC and two ipsilateral or locoregional recurrences. Although this observation is limited by the small sample size and cannot support specific surgical recommendations, it may be relevant to individualized surgical planning and counseling of *POT1* PV heterozygotes.

Early detection of BC is associated with improved outcomes and may reduce treatment intensity. According to the NCCN guidelines, annual breast MRI as part of a surveillance plan is indicated for women with lifetime BC risk ≥ 20% [21]. Until recently, there were no published guidelines for surveillance of *POT1* PV carriers. An expert opinion publication recommended a comprehensive physical examination including lymph nodes and skin, as well as an annual blood count. In some cases with significant family history of cancer, an annual whole body MRI was recommended, with the addition of brain MRI in families with glioma cases [5]. None of the available recommendation sets, including the recently published UK guidelines, mention or provide evidence to recommend enhanced breast surveillance for *POT1* PV heterozygotes [22]. Based on the findings in this study, annual breast MRI screening may be considered in *POT1* PV heterozygotes, particularly in carriers of the Ashkenazi Jewish c.233T > C founder allele. The initiation of screening may be later than that recommended for *BRCA* PV heterozygotes, given that none of the affected patients in this cohort was diagnosed before age 40. In analogy to NCCN strategies for women with lifetime BC risk ≥ 20%, screening could be initiated 10 years earlier than the youngest BC diagnosis in the family.

The impact of *POT1* PVs on BC risk remains uncertain. Available evidence does not support bilateral risk reducing mastectomy for unaffected carriers based on *POT1* status alone. Surgical risk reduction should therefore be considered only in the context of individualized risk assessment, including personal and family history, breast imaging findings, and patient preference.

Incorporation of *POT1* PVs into formal risk assessment models is, at present, premature. Melanoma was observed in only 5 of 13 carriers, and in 2 cases it was diagnosed with or after, rather than before the occurrence of BC. We advocate for inclusion of *POT1* in comprehensive germline cancer predisposition panels, rather than restricting testing to melanoma focused panels.

The main limitations of this study are its small sample size and incomplete clinical information in some cases. The association between *POT1* PVs and BC risk requires validation through larger studies. Future research should aim to clarify the penetrance of *POT1* PVs and define the clinical features in this population, to guide tailored surveillance strategies. Collection of long-term prospective data, including BC outcomes among carriers of different *POT1* PVs, is critical to defining cancer risk and guiding evidence-based clinical recommendations.

## Data Availability

The datasets generated in the current study are not publicly available but could be made available upon request from the authors.
